# Post-process optimization of 3D printed poly(lactic-co-glycolic acid) dental implant scaffold for enhanced structure and mechanical properties: effects of sonication duration and power

**DOI:** 10.1007/s10856-021-06561-3

**Published:** 2021-07-31

**Authors:** R. N. V. C. Virinthorn, M. Chandrasekaran, K. Wang, K. L. Goh

**Affiliations:** 1 Newcastle University in Singapore, SIT@NYP, 172A Ang Mo Kio Avenue 8 #05-01, Singapore, 567739 Singapore; 2 Newcastle Research & Innovation Institute Singapore, 80 Jurong East Street 21, #05-04, Singapore, 609607 Singapore; 3 Emirate Nuclear Technology Center (ENTC), Department of Chemical Engineering, Khalifa University of Science and Technology, Abu Dhabi, United Arab Emirates PO Box 2533,

## Abstract

We described a technique of a post-process stage to partially remove the poly(vinyl alcohol) (PVA) binder in Poly(lactic-co-glycolic acid) (PLGA) dental scaffolds. The scaffolds were exposed to ultrasonic waves while immersed in an ethanol/acetone solvent mixture that possessed both polar and nonpolar properties. A factorial experiment was conducted in which the scaffolds were treated to three levels of sonication power (*p*_W_): 0, 20% (22 W) and 40% (44 W), and soaking duration (*t*): 5, 15, and 30 min. The treated scaffolds were characterized by FT-IR, optical microscopy, and mechanical (compressive) testing. FT-IR revealed that the amount of PVA decreased with increasing *p*_W_ and *t*. Two-way ANOVA revealed that increasing *p*_W_ and *t*, respectively, resulted in increasing scaffold surface area to volume (SVR). Sonication and solvent caused structural damage (i.e., unevenness) on the scaffold surface, but the damage was minimal at 20% *p*_W_ and 30 min. The optimal values of p_W_ and t resulting in enhanced fracture strength, strain and toughness were 20% and 30 min, respectively, which corroborated the findings of minimal structural damage. However, sonication had no significant effects on the scaffold stiffness. Mechanistic analysis of the effects of sonication predicted that the ultrasonic energy absorbed by the scaffold was sufficient to disrupt the van Der Waals bonds between the PVA and PLGA but not high enough to disrupt the covalent bonds within the PLGA. This technique is promising as it can partially remove the PVA from the scaffold, and mitigate problematic issues down the line, such as thermal degradation during sterilization, and undue delay/variability in biodegradation.

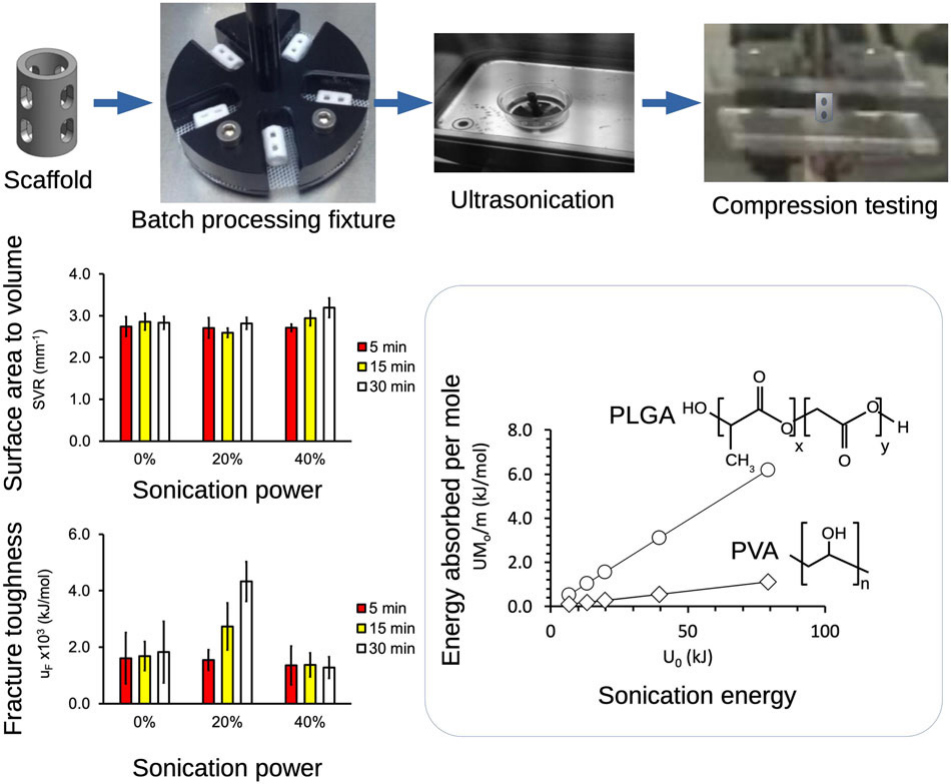

## Introduction

Preserving the natural contour of the alveolar ridge after a tooth extraction process, known as dental socket preservation, is important as it can facilitate future reconstruction with an implant prothesis. This can be achieved by inserting fillers, such as grafts or synthetic scaffolds, into the tooth-extracted site to prevent the alveolar ridge from collapsing [1], thus retaining the natural contour of the alveolar ridge as well as minimizing bone lost by bone resorption [2]. Of late, porous and moldable synthetic scaffolds made from poly lactic-co-glycolic acid (PLGA) [3–6] are popular alternatives to dental bone grafts [7] for dental socket preservation. As an implant, PLGA-based scaffolds are biocompatible with the human body [3–6]. Its biodegradation rate and mechanical properties can also be tailored by varying the ratio of its copolymers, namely lactic acid and glycolic acid, which are bonded together via ester linkages [4, 5, 8]. For dental application, one advantage of using such a synthetic implant is that the scaffold in the socket could be flushed to the level of the base of the bone [3, 4].

In powder-based additive manufacturing processes, such as selective laser sintering and inkjet printing, the forming of polymer-based scaffolds would require a binder for binding the powders [9]. With regard to the manufacturing of the PLGA scaffolds by inkjet printing, Poly(vinyl alcohol) (PVA) has been identified as a binder for the PLGA powders [8, 10–12]. It acts as a surfactant to emulsify, stabilize the dispersed state of PLGA and, consequently, maintain the scaffold structural integrity during the processing stage. Binding the PLGA powders in this way can result in a structure that possesses the resultant strength and stiffness of the scaffold which are compatible to those of alveolar bone [7]. Different methods of forming PLGA scaffolds using PVA as a binder have been reported, such as melt-molding particulate leaching to produce sponge scaffold [8], wet spinning to produce hollow (porous-wall) fiber scaffold [10] and emulsion electrospinning to produce nanofibrous mat-like scaffolds [12]. For details of previous studies related to the current paper, see Table SI—1 in Supplementary Information for a summary of the findings of the mechanical properties and structure of PLGA-based scaffold that utilized PVA as a binder.

The use of PVA as a binder for facilitating the forming of the porous PLGA scaffold presents several problematic issues for the scaffolds. First, during dry heat sterilization (whereby the temperature could go up to 160 °C or higher) [13, 14], a proportion of the PVA could thermally degraded to produce polyene (yellowish) residues, resulting in decolorization of the scaffold [15–18]. While this is esthetically unacceptable from the dental perspective, the thermally degraded PVA exhibits lower fracture strength and strain [19]. Consequently the lower fracture strength and strain could reduce the overall structural integrity of the scaffold, as well as increase the variability in the final mechanical property. This presents a hurdle to achieve the desired mechanical properties of the scaffold. The second issue is related to biodegradability. Ideally, the scaffold should provide an adequate duration for the alveolar bone to regenerate as the scaffold biodegrades. Although the PLGA scaffold is suited for this purpose, the PVA present in the scaffold may have lower degradation rate and this could result in undue delay in degradation. The extent to which the PVA degrades depends on the degree of hydrolysis involved in the production of the PVA [8]. In addition, the PVA residuals formed an interconnected network with the PLGA and this could hinder the degradation of the PLGA [11]. The rate of diffusion of the surrounding body fluids into the PLGA could decrease and, consequently, this decreases the speed of degradation and further pose a mismatch between the scaffold degradation rate and bone tissue regeneration rate. Again, this presents a challenge to meeting regulatory requirements for the product to state the biodegradation time.

Ideally, one may wish to implement a post-processing stage that can cause the PVA to leach or to be removed by a solvent, while the PLGA remains inert to the solvent or else possesses a low dissolution rate in the solvent. One might wish to optimize the PVA concentration through such a post-process approach to achieve a scaffold with the desired mechanical property with minimal disruption to the overall structure.

From the current understanding of the use of shock waves generated by cavitation (during sonication) for breaking down powders into nanoparticles [20], we proposed a novel approach to use ultrasonic waves to dislodge as much PVA residuals as possible in the PLGA-based scaffold, in the presence of a solvent mixture (possessing both polar and nonpolar properties). The main purpose of the polar-nonpolar solvent mixture was to facilitate the dissolution and re-bonding of PLGA particles within the scaffold so as to minimize the diminution of the structural integrity of the final PLGA scaffold. However, the sonication and chemical effects on the structure and mechanical properties of the PLGA scaffold are not clearly understood. Here, we investigated the effects of sonication power, *p*_W_, and soaking duration, *t*, on the mechanical properties and structure of the PLGA/PVA scaffolds and the optimal levels for the *p*_W_ and *t* for the PLGA scaffolds to achieve enhanced mechanical properties.

## Materials and method

### Theory

As ultrasonic waves produced by the ultrasonic machine propagate through the ethanol/acetone medium containing the PLGA scaffolds, this would generate cyclical high and low pressures throughout the medium [20]. At low pressures (rarefaction), microscopic vapor bubbles (cavitation) were formed; at high pressures (compression), the bubbles collapsed and produced shock waves of high energy, in the form of microscopic jet stream, traveling at high velocity, with pressure of the order of hundreds of mega pascals and temperatures higher than the *T*_bath_. The total amount of energy, *U*_0_, generated by an ultrasonic machine is related to the *p*_W_ and *t*, according to

1 $${{{{U}}}}_0 \,=\, {{{{p}}}}_{{{{{\mathrm{W}}}}}}{{{{t}}}}$$

[20]. This energy is capable of dispersing nanometer size particles. According to this relationship, two PLGA/PVA scaffolds treated at a given p_W_, for two different ts, could lead to different degree of removal of PVA. Similarly, two PLGA/PVA scaffolds treated at a given *t*, for two different *p*_W_s could also lead to different degree of removal of PVA. Now, the *U* would be partially attenuated (the energy would be dissipated into the solvent mixture) as the ultrasonic wave propagated through the solvent mixture toward the scaffold. Consider an ultrasonic source generating an acoustic intensity of *I*_0_ (W/m^2^), at a given distance (x) from the source. The acoustic intensity, *I*, is given by

2 $${{{{I}}}} \,=\, {{{{I}}}}_0\exp ( - 2\alpha {{{{{\mathrm{x}}}}}}),$$

where the absorption coefficient

3 $${\upalpha}\,\sim\, {{{\mathrm{G}}}}\mu {{{\mathrm{f}}}}^2/{\upgamma}$$

μ represents the dynamic viscosity (kg m^−1^ s^−1^), γ is the density of the solvent mixture, and G is a constant [20]. The nature of G may be understood by noting that both G and μ, when combined as Gμ, can be identified as the effective dynamic viscosity where G serves as a weighting factor to modulate μ. According to Taurozzi et al. they have assigned *G* = 2/3. This is not based on a detailed analysis of the viscosity of the liquid but—with a numerical value less than unity—it indicates that the weighting factor provides the lower limit of μ.

As the waves collapse, how this result in breaking the PLGA powders would depend on the sonication energy [20]. At low energy, this could disperse the powder agglomerates which were bound by van Der Waals forces; at higher energy this could break up the individual particles, which were bound by chemical bonds [20]. Here, Eqs. (1), (2), (3) provide the means to estimate this effect.

### PLGA scaffolds

**Fig. 1:**
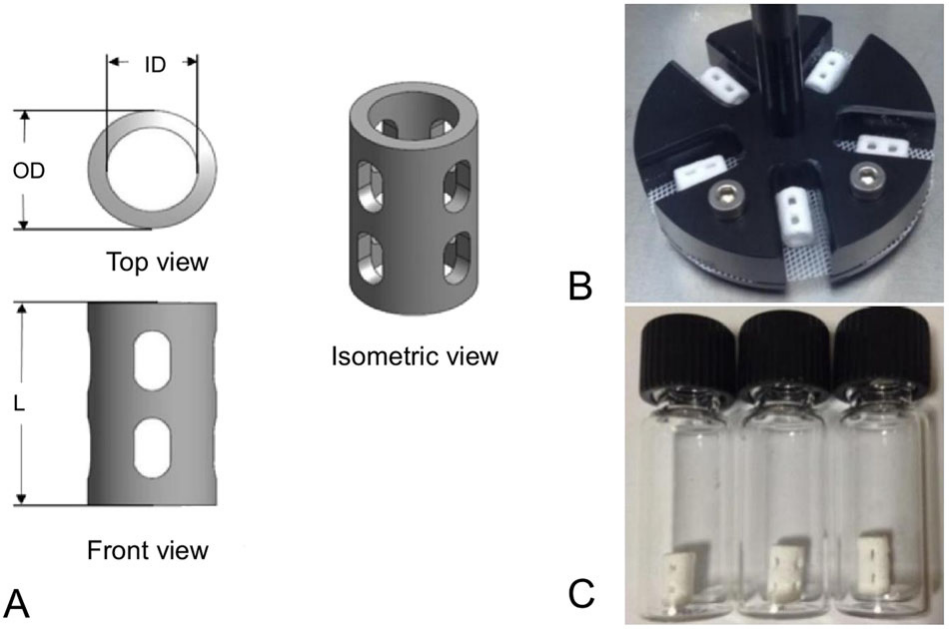
PLGA/PVA scaffolds. **A** Schematics of a scaffold showing the top, isometric and side view. The outer diameter (*D*_OD_), inner diameter (*D*_ID_), length (*L*_0_), and total surface area (*A*_s_) of the printed scaffold were 3.6 mm, 2.0 mm, 6.5 mm and 126.0 mm^2^, respectively. **B** Scaffolds held in a customized jig used for agitation during sonication. **C** Scaffolds after sonication

Figure 1A shows schematics of a PLGA/PVA scaffold. The design specifications required that the dimensions of the scaffold were as follows: outer diameter (*D*_OD_) = 3.6 mm; inner diameter (*D*_ID_) = 2.0 mm; length of the scaffold (*L*_0_) = 6.5 mm. The 3D printed PLGA/PVA scaffolds were purchased from M/s Bio-scaffold International Pte Ltd (BSI, Singapore). These were patented PLGA-based dental scaffolds, known otherwise as Bioscaff Alvelac [7]. According to BSI, the PLGA was purchased from Corbion (PURAC, www.corbion.com). The PLGA possessed a monomer ratio of lactic acid to glycolic acid of 85:15. The PVA was purchased from Merck Pte. Ltd. (affiliate of Merck KGaA, Darmstadt, Germany, www.merckmillipore.com).

BSI used a Z-Corp 310 Plus 3D printer (www.3dsystems.com, previously under Z-Corp, Burlington, USA) to print the scaffolds. This printer had a main features resolution of 300 × 450 dpi and vertical-build speed of 1.0 inch/h (25 mm/h). While the printer could accommodate a maximum build size of 8 × 10 × 8 inches (203 × 254 × 203 mm), the size of each PLGA scaffold to be produced was much smaller than the maximum size capacity. Thus it was possible to produce multiple scaffolds (<80) in a single run with a duration of about 20 min per run.

Details of the exact formulation of the blended mixture of PLGA and PVA that led to the final product, i.e., the scaffold, were proprietary information of the PLGA scaffold manufacturing company and were not made available to us. We have been informed by the company that the scaffolds were made from blending milled PLGA and PVA followed by 3D printing. According to information gathered from a published patent document [7], PLGA granules were milled at a frequency of about 30 cycles to yield a particle sizes below 300 μm and suitable sizes were selected for printing the scaffold. The PLGA scaffold manufacturing company had informed the authors that the volume ratio of PVA:PLGA ranged 2:8–7:10, and they could not be more specific given that the exact composition of the PVA and PLGA was proprietary information. We have attempted to provide an estimate of the composition of the components of the PLGA-based scaffold for informational purpose. The starting point of our calculation was based on the volume ratio information provided by the PLGA scaffold manufacturing company, informed by the basic design concepts of composite materials and foams from the patent document [7]. For the benefit of the reader, in Section 2 of Supplementary Information, we have provided the details of our approach to arrive at simple order-of-magnitude estimates of the composition of the PLGA-based scaffold. We reported that the volume fractions of PVA and PLGA used were 0.4 and 0.6, respectively, valid to order of magnitude. This yielded a volume ratio of 2:3 which falls within the range of values of the volume ratio provided by the manufacturer.

### Post-processing by sonication

The acetone (>99.5% purity) and ethanol (~95% purity) were purchased from Sigma Aldrich (www.sigmaaldrich.com). Both acetone and ethanol (which contain polar and nonpolar groups) were mixed to produce a solvent for the scaffold post-processing study, for the purpose of dissolving polar (e.g., water, PVA) and other nonpolar molecules. The method for the preparation of the polar-nonpolar solvent mixture was proprietary information. In short, a starting amount (also proprietary information) of the respective components of the acetone/ethanol solvent was developed first, following by varying the amount of the ethanol, to establish the range of *p*_W_ and *t* to be used in the current study. This led to a feasible scaffold with reduced amount of PVA present, adequate mechanical properties and adequate surface to volume ratio. Importantly, this was done by selecting values of *p*_W_ and *t* within the extremes of practical values, though some limitations were placed on the values of *p*_W_ to ensure that the scaffold experienced moderate depolymerization of PVA [21], as explained in Section 2.1.

Careful consideration of the choice of the values of p_W_ would be required in order to moderate the disruption of the PVA network and dislodge the PVA from the PLGA, with minimal effect on depolymerization and disruption to the bulk of the PLGA. It has been reported that an input power of 10 W could result in depolymerization of PVA to a small extent (about 10%) [21]. At a higher input power, namely 200 W (i.e., the upper limit of their study), the PVA in solution experienced extensive depolymerization [21]. Thus, there exists a threshold power related to the formation of cavitation (i.e., the cavitation threshold) below which depolymerization of PVA in solution will not occur. Unfortunately, the precise cavitation threshold is still not known. Thus, in this study, the values of p_W_, namely 20% (22 W) and 40% (44 W) were chosen as these represented the intermediate values of the two extremes (10 W and 200 W).

The ultrasonic machine (Elma Transonic T 490 DH) used in this study had the following specifications: ultrasonic frequency of 40 kHz, total power consumption of 330 W, and *p*_W_ (RMS) of 110 W (at 100%) and 130 W (at 140%). The tank internal dimensions were 240 mm × 137 mm × 100 mm. During sonication, the scaffolds (in the beaker) were placed at the center of the tank.

A factorial experiment was conducted in which the scaffolds were treated to three levels of *p*_W_ namely 0 (i.e., no sonication), 20% (22 W), 40% (44 W), and three levels of *t*, namely 5, 15, and 30 min. This yielded a series of experimental runs that corresponded to different combinations of *p*_W_ and *t* using Eq. (1), by setting values of *p*_W_ = 22 W (i.e., 20%) and 44 W (i.e., 40%) at the respective *t* levels, namely 5, 15, and 30 min.

For the factorial experiment the PLGA/PVA scaffold specimens were grouped according to the respective combinatorial levels of the *p*_W_ and *t.* Five specimens were used for each combination of levels of treatment. A solvent mixture containing 100 ml of ethanol and acetone (the exact amount was proprietary information) was prepared in a beaker and magnetically stirred at 250 rpm for 15 min. The specimens were held in a customized jig which could accommodate 5 scaffolds at a time (Fig. 1B) and the jig was introduced into the beaker (containing the solvent mixture). Sonication began after the beaker was placed in a water bath in the ultra-sonication machine. Thereafter, the scaffolds were removed from the solvent and a simple mechanical process was applied to extract water and ethanol/acetone droplets that were present in the scaffolds by gently pressing and pin-rolling the scaffolds on a flat Teflon mesh. Water and ethanol/acetone were the key components necessary for PVA binding with PLGA during the 3D printing process. However, the presence of water and ethanol/acetone in the PLGA (entrapped in closed pores) after forming could produce the following results: (1) the generation of residual pressure within closed pores; (2) incomplete bonding of PLGA particles; (3) unintended degradation during the sterilization step. Removing the excess water and ethanol/acetone droplets was a key step following the forming stage. Figure 1C shows a photo of the scaffolds at the end of the post process.

### Scaffold dimensions

After the sonication treatment, prior to mechanical testing, the *D*_OD_, *D*_ID_, and *L*_0_ of the scaffold were measured using a conventional optical microscope (Olympus BX51M model). In addition to the measurement of scaffold dimensions, microscopic examinations of the scaffold surfaces were also performed using the optical microscope.

Measurements were carried out at four sites along the scaffold axis to account for non-uniformity. With regard to the *D*_ID_, measurements were carried out at the two end faces of the scaffold. To ensure consistency in the measurements of the *D*_OD_ and *L*_0_, the scaffold was placed horizontally on a V shape groove jig, mounted on the microscope stage. To measure *D*_ID_, each specimen was vertically positioned on a microscopic slide. With regard to *L*_0_, measurements were carried out at four sites around the scaffold. The measured values were averaged to obtain representative values of *D*_OD_, *D*_ID_, and *L*_0_, respectively. The cross-sectional area (A) of the specimen was estimated by setting *A* = π[*D*_OD_^2^ − *D*_ID_^2^]/4.

The *A* and *L*_0_ were used in the computation of the stress and strain from the force-displacement data obtained from compressive testing (Section 2.6).

In addition, the ratio of surface area to volume (SVR) of the scaffold was determined as follows. The surface area of the scaffold (*A*_S_) was estimated by summing the areas of the outer and inner surfaces and the *A* at the two end faces. The volume of the scaffold was estimated by *AL*_0_. Thus the SVR was estimated to order of magnitude by *A*_S_/*AL*_0_.

### Fourier Transform Infrared (FT-IR) spectroscopy

Fourier transform infrared (FT-IR) spectroscopic analysis of the composition of the PLGA-based scaffold was carried out on the pieces of crushed scaffolds. FT-IR was performed using a Brucker Vertex 80 v spectrometer with a parallel beam transmittance accessory. Using the on-board camera of the spectrometer, surfaces with a high degree of evenness were located through the display screen to ensure optimal interaction of the IR radiation with the specimen. Then each transmittance spectrum was recorded from 1000 to 3000 cm^−1^ using the following input settings: pressure 250 Pa, resolution of 4 cm^−1^ and 500 scans per specimen.

### Mechanical tests

Compression tests were carried out on a mechanical tester (Shimazu AGS-X). A 10-kN loadcell was used to measure the force generated in the specimen while it was subjected to compressive loading. The scaffolds were compressed in the direction perpendicular to the circular face of the cylindrical samples at a rate of 5 mm/min until the scaffold was crushed.

**Fig. 2:**
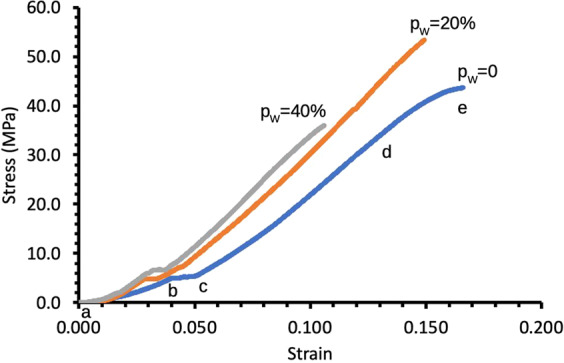
Stress–strain curves of the PLGA/PVA scaffolds were derived from compression testing. These were obtained for *p*_W_ = 0, 20, and 40% at *t* = 30 min. The region between points a to b indicates the region of initial loading. The region between points b to c indicates a characteristic plateau, suggesting possible collapse of the engineered holes in the scaffold (Fig. 1A). The region between points c to d accounts for the mechanical support provided by the PLGA-based material to resist the load. Point e indicates the point of fracture

Stress–strain data was derived from the force-displacement data of each specimen. Figure 2 shows a graph of typical stress versus strain curves corresponding to PLGA/PVA scaffolds treated at three different *p*_W_ values. Stress was calculated using *σ* = *F*/*A*, where *F* represented the force generated in the specimen during compression, and *A* the original cross-sectional area of the scaffold that was in contact with the platens of the mechanical tester. Strain was calculated using *ε* = Δ/*L*_0_, where Δ represented the displacement during compression.

The compressive fracture strength (*σ*_U_), strain at fracture (*ε*_U_), stiffness (*E*), and fracture toughness (*u*_F_) were determined for each specimen. The *σ*_U_ was associated with the maximum stress of the stress–strain curve; the corresponding strain point parameterized the *ε*_U_. The E was determined from the gradient of the linear region of the stress–strain curve, which was located in between point c and d, where the external load was predominantly resisted by the bulk material. The *u*_F_ was determined from the area under the stress–strain curve from the origin to *ε*_U_.

### Statistical analysis

Statistical analysis was carried out to evaluate the effects of the *p*_W_ and *t* on the scaffold structure and mechanical properties using software (RStudio, Version 1.2.5033). Following checks to ensure that the data satisfied the conditions of homogeneity of variance of the residuals and normality of residuals, a one and two-way (two-tailed) analysis of variance was used to evaluate our results at an alpha level of 0.05. A total of 45 scaffolds were tested and used for the statistical analysis. The number of test specimens for each treatment was 5. Results were expressed as mean ± standard deviation (SD).

## Results

### Microscopic examination

As obtained PLGA scaffolds showed a characteristic white surface (Fig. 1C). Treating the specimens to soaking durations of 5, 15, and 30 min, at the respective sonication power (i.e., *p*_W_ = 0, 20, and 40%), did not result in appreciable changes in the color texture.

**Fig. 3:**
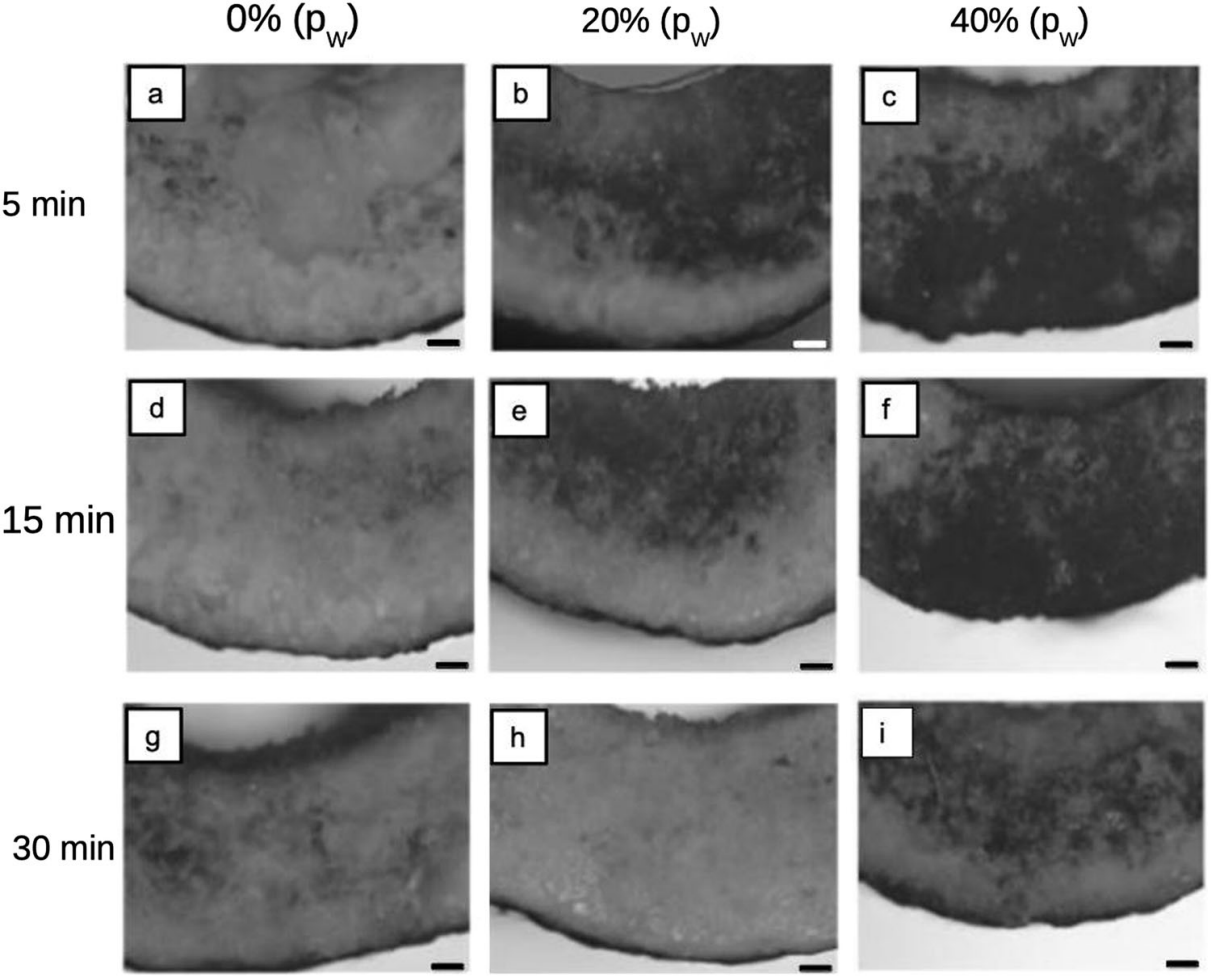
Optical micrographs of PLGA/PVA scaffold treated to the respective sonication power levels, 0, 20 (*p*_W_), and 40% (*p*_W_), at 5, 15, and 30 min soaking duration. Here, each panel (a to i) shows the surface of a PLGA specimen, corresponding to a specific treatment level. For instance, the image in panel a was derived from a specimen treated at *p*_W_ = 0% and *t* = 5 min. Scale bar indicates 100 μm

When sonication was applied to the scaffolds at 5, 15, and 30 min, optical microscopy revealed large areas of irregular patches as seen in Fig. 3. Depending on the *p*_W_ level, scaffolds could appear as partially darkened, i.e., some regions of the surface appeared darker than others; other scaffolds appeared completely darkened. These were attributed to unevenness of the surface, following dislodging of PVA, and/or dissolution and re-bonding. Figure SI—1 in the Supplementary Information section provides additional examples (macroscopic and microscopic views) to illustrate the unevenness. Thus the uneven scaffold surface could be observed as a result of the following effects: (1) parallel light rays incident onto the scaffold surface were scattered randomly leading to diffuse reflection; (2) limitation in the depth of field so that extended undulated layers arising from particle (300 μm) re-bonding were located beyond the depth of field. On that note, the scaffolds least affected were those treated to *p*_W_ = 20% and *t* = 30 min.

### Fourier transform infrared (FT-IR) spectroscopy

To evaluate the extent of the PVA present in the PLGA-based scaffold, the FT-IR spectra were analyzed for changes in the intensity of the absorption bands of the PLGA-based scaffold that could be attributed to the changes in the respective PLGA and PVA.

**Fig. 4:**
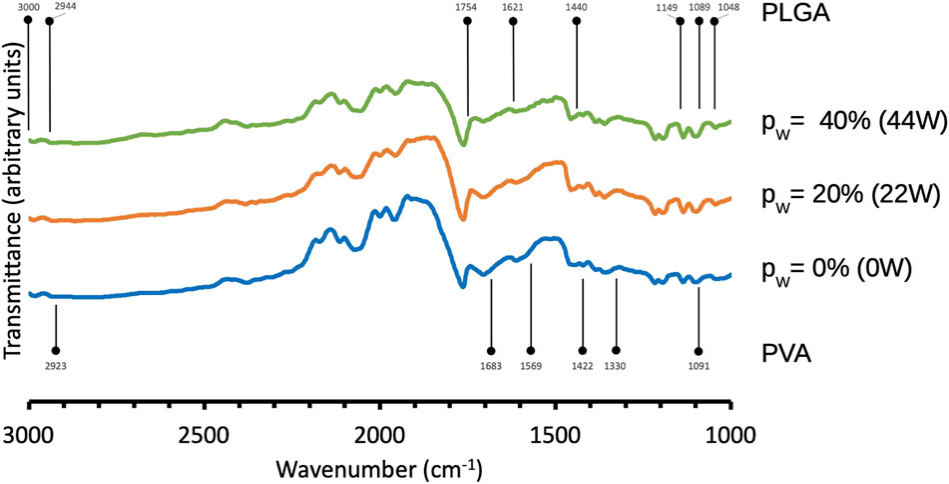
Fourier transform infrared spectra of PLGA/PVA scaffold treated to the respective sonication power levels, 0, 20 (*p*_W_), and 40% (*p*_W_), at 5 min soaking duration. Of note, PVA and PLGA have linear formula given by (CH_2_CH(OH))_n_ and (C_3_H_4_O_2_)_x_(C_2_H_2_O_2_)_y_ respectively

Figure 4 shows typical FT-IR spectra of PLGA/PVA scaffolds corresponding to *p*_W_ = 0, 20, and 40% at *t* = 5 min. The wavenumbers labeled on the graphs corresponded to “mid-point” value of range of wavenumbers reported in the literature for the respective PLGA and PVA material. The key absorption bands of the PVA overlapped those of the PLGA in the high wavenumber region (3000–2923 cm^−1^), mid-range region (1754–1330 cm^−1^), and low wavenumber region (1149–1048 cm^−1^). Separately, we have identified three regions of interest that revealed reductions in the PVA content in the PLGA-based scaffold. The first region corresponded to the wavenumbers between 1740 and 1569 cm^−1^. It showed that the intensity of the bands decreased appreciably with increase in *p*_W_. These decrease were attributed to the diminishing population of the C=O and C–O (stretching mode) from the acetate group of the PVA (in the presence of the poly (vinyl acetate) (PVAc), owing to possible incomplete hydrolysis in the polymerization process to obtain PVA, resulting in a mixture of PVA/PVAc [22]). The second region corresponded to the wavenumbers between 1422 and 1330 cm^−1^; it showed that the intensity of the bands decreased (albeit slightly) with increase in *p*_W_. These reduction in intensity were attributed to the diminishing population of the C–H (bending vibration of CH_2_ and deformation vibration) from the PVA. The third region corresponded to the wavenumbers between 1149 and 1048 cm^−1^. In this region, at *p*_W_ = 0, the vibration bands arising from C–O stretching (PVA) were masked by those of C–O–C, C–O, and C–CH_3_. As the p_W_ increased, this resulted in an overall narrowing of the band due to the reduction in population of C–O from the PVA. Concomitantly, the effect from the C–O–C, C–O, and C–CH_3_ (PLGA) became more dominant and thus the bands appeared more pronounced at higher *p*_W_. Table 1 provides further details about these absorption bands.

**Table 1:** Summary of band frequencies corresponding to PVA and PLGA, derived from FT-IR of PLGA/PVA scaffold

Polymer	Wavenumber^a^ cm^−1^	Chemical bonds	References
PLGA	3000–2996 (2998)	CH_2_ asymmetric stretching	[27–29]
	2962–2922 (2943)	CH_2_ asymmetric stretching	[12, 27–29]
PLGA	1759–1747 (1742)	C=O stretching	[12, 27–32]
PLGA	1620	C–O	[28]
PLGA	1454–1421 (1438)	C–H stretching	[12, 28, 29, 31, 32]
PLGA	1392–1363 (1377)	O–H bending	[27–29, 32]
PLGA	1182–1113 (1147)	C–O–C stretching, C–O stretching	[12, 27–29, 32, 33]
PLGA	1093–1082 (1087)	C–O stretching vibration from ether group	[28, 29, 32]
PLGA	1047–1046 (∼1046)	C–CH_3_	[29, 32]
PVA	2924–2917 (2921)	C–H stretching	[34, 35]
PVA	1714–1650 (1682)	C=O and C–O stretching from acetate groups	[34–36]
PVA	1568	C=O stretching from acetate groups	[37]
PVA	1425–1418 (1422)	C–H bending vibration of CH_2_	[34, 37]
PVA	1334–1324 (1329)	C–H deformation vibration	[34, 37]
PVA	1099–1081 (1090)	C–O stretching of acetyl groups	[34–38]

^a^Figure in parenthesis denotes the mid-point value of the range of wavenumbers

More importantly, as the absorption bands at 1433, 1330, and 1091 cm^−1^ corresponding to PVA were still visible at *p*_W_ = 20 and 40%, this suggested that the PVA was still presence in the scaffold, albeit in reduced amount. In Section 4 of the Supplementary Information, we presented a description of our approach to apply models based on the classic Beer–Lambert law, to compute order of magnitude estimates of the volume fraction of PVA residue present in the sonicated scaffold. Recalled that the volume fraction of PVA present in the pristine scaffold was estimated at 0.4 (Section 2.2). We predicted that, to order of magnitude, the volume fraction of PVA decreased to (i) 0.31 (*p*_W_ = 20%) and (ii) 0.26 (*p*_W_ = 40%). Thus the amount of PVA residue present in the sonicated scaffold was 0.78 (*p*_W_ = 20%) and 0.66 (*p*_W_ = 40%) times smaller than that of the pristine scaffold. Overall, this indicated a progressive decrease in PVA with increasing *p*_W_.

### Scaffold surface area to volume ratio

Two-way ANOVA showed that the *P* values for p_W_ (*P* = 0.003) and *t* (*P* = 0.005) were both very small, indicating that there was very strong evidence that both factors affected SVR. The *P* value for the interaction (*P* = 0.053) was greater than the alpha level, showing that there was no evidence for an interaction between the factors. The main effects may be interpreted independently of one another: sonication power affects SVR as does only soaking duration.

**Fig. 5:**
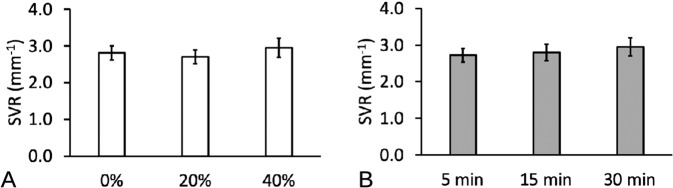
Main effects plots of the surface area to volume ratio (SVR) of PLGA/PVA scaffold with respect to (**A**) sonication power, *p*_W_, and (**B**) soaking duration, *t*. Note that the value of the respective bars on each graph represented the average (plus–minus standard deviation of sample) of all the levels at each *p*_W_ or at each *t*

**Fig. 6:**
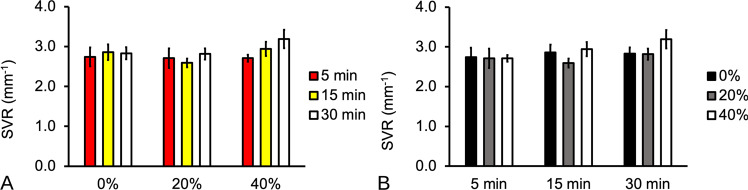
Interaction plots of the surface area to volume ratio (SVR) of PLGA/PVA scaffold with respect to (**A**) sonication power, *p*_W_, and (**B**) soaking duration, *t*

The overall effects may be summarized by the main effects plot for *p*_W_ and *t* (Fig. 5) which showed that increases in sonication power and soaking duration resulted in increases in SVR. For completeness, the details of the variation of SVR with respect to t at each level of p_W_ and vice versa are shown in the interaction plots (Fig. 6). Thus, the main effects plots of SVR versus *p*_W_ showed that at lower sonication power, i.e., *p*_W_ = 20%, changes in *t* did not lead to an appreciable change to the SVR (Fig. 6A). At higher sonication power, i.e., *p*_W_ = 40%, the SVR increased with increasing *t* (Fig. 5A). Similar trends were also observed for the case of SVR versus *t* (Fig. 5B and Fig. 6B). Overall, these results indicated that the scaffold volume could be drastically reduced compared to the surface area. The results of the SVR are summarized in Table 2. The mean SVR of untreated specimens was found to be 2.81 ± 0.11 mm^−1^—this could be regarded as the baseline.

**Table 2:** Summary of results from compression testing of PLGA/PVA scaffolds

*p*_W_ (%)	*t* min)	*U*_0_ (kJ)	SVR (mm^−1^)	*E* (MPa)	*σ*_U_ (MPa)	*ε*_U_	*u*_F_ × 10^3^ (kJ/m^3^)
Untreated	–	–	2.81 ± 0.11	171.9 ± 53.0	22.3 ± 5.8	0.131 ± 0.009	1.45 ± 0.32
0	5	0	2.74 ± 0.24	335.0 ± 78.9	36.4 ± 12.8	0.108 ± 0.021	1.61 ± 0.91
0	15	0	2.86 ± 0.20	285.2 ± 47.8	34.9 ± 7.7	0.122 ± 0.015	1.69 ± 0.51
0	30	0	2.83 ± 0.15	231.3 ± 62.0	32.6 ± 13.7	0.137 ± 0.032	1.83 ± 1.09
20	5	6.6	2.71 ± 0.24	304.6 ± 71.6	35.2 ± 7.9	0.117 ± 0.014	1.54 ± 0.36
20	15	19.8	2.59 ± 0.11	287.7 ± 18.4	43.3 ± 5.7	0.151 ± 0.020	2.74 ± 0.83
20	30	39.6	2.82 ± 0.14	341.3 ± 13.9	58.4 ± 4.0	0.171 ± 0.015	4.33 ± 0.70
40	5	13.2	2.71 ± 0.09	284.3 ± 59.5	31.6 ± 11.0	0.110 ± 0.021	1.36 ± 0.68
40	15	39.6	2.94 ± 0.18	278.7 ± 47.6	31.2 ± 6.6	0.112 ± 0.013	1.38 ± 0.42
40	30	79.2	3.19 ± 0.23	286.6 ± 57.7	30.3 ± 6.9	0.106 ± 0.013	1.28 ± 0.38

### Effects on elasticity and fracture of scaffold

**Fig. 7:**
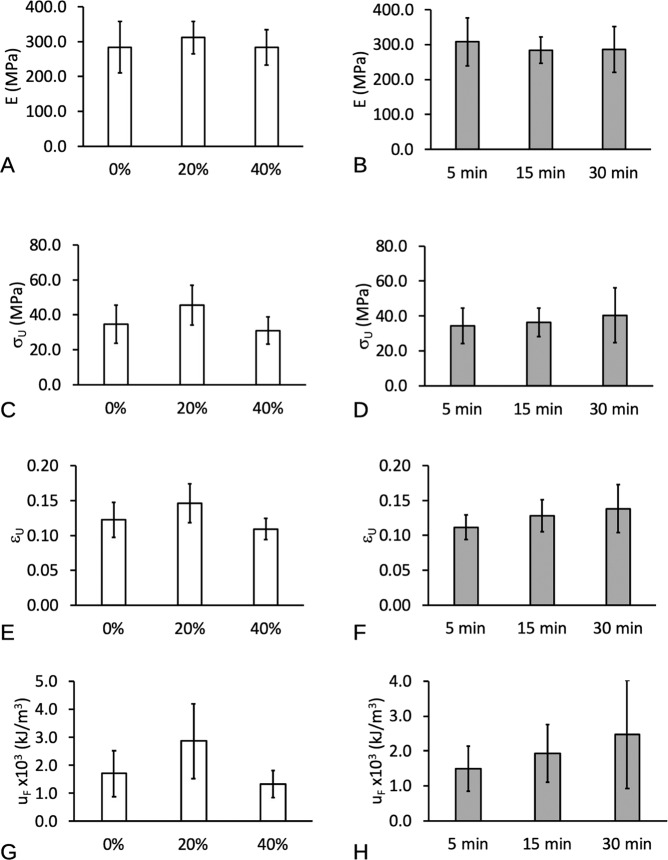
Main effects plots of the mechanical properties of PLGA/PVA scaffold with respect to sonication power (*p*_W_), and soaking duration (*t*). **A**, **B** Stiffness, **E**. **C**, **D** Fracture strength, *σ*_U_. **E**, **F** Strain at fracture, *ε*_U_. **G**, **H** Fracture toughness, *u*_F_. Note that the value of the respective bars on each graph represented the average (plus–minus standard deviation of sample) of all the levels at each *p*_W_ or at each *t*

**Fig. 8:**
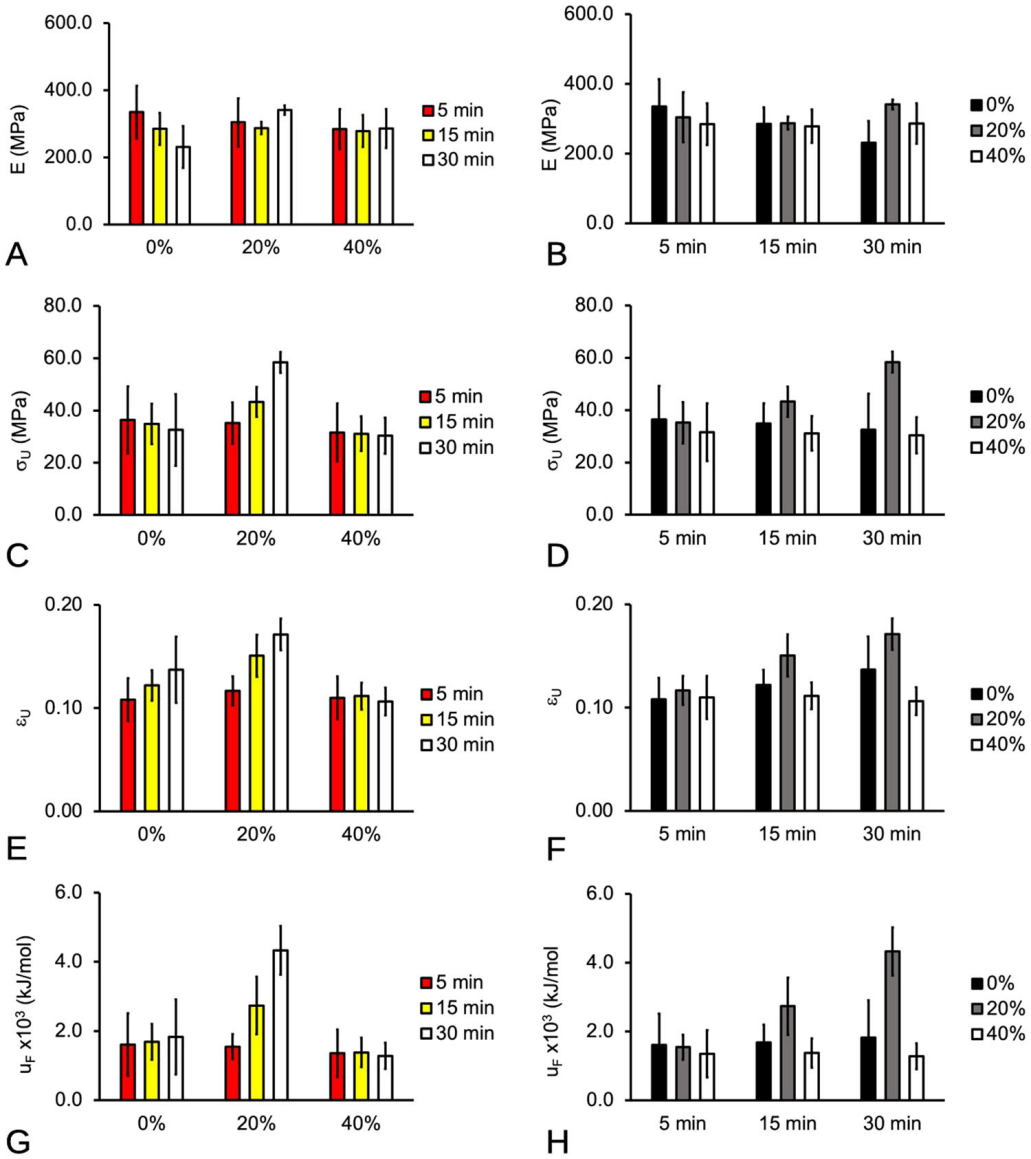
Interaction plots of the mechanical properties of PLGA/PVA scaffold with respect to sonication power (*p*_W_), and soaking duration (*t*). **A**, **B** Stiffness, **E**. **C**, **D** Fracture strength, *σ*_U_. **E**, **F** Strain at fracture, *ε*_U_. **G**, **H** Fracture toughness, *u*_F_

In this section the results of the effects of p_W_ and t on the mechanical properties of the scaffold were presented for the main effects (Fig. 7) and the individual (interaction) effects (Fig. 8). Here, the main effects described the magnitude of the mechanical property at each level of *p*_W_ (averaged over the variation due to *t*) and at each level of *t* (averaged over the variations due to *p*_W_). The individual effects described the variation in the magnitude of the mechanical property in the presence of varying *t*, at each level of *p*_W_, and vice versa for *p*_W_ at each level of *t*. For details of the respective mechanical properties, see Table 2.

With regard to the main effects of the respective *p*_W_ and *t* on *E*, the *P* values, i.e., 0.2922 and 0.4266, were both greater than the alpha level, showing that there was no evidence that both factors affect *E*. The *P* value for the effects arising from the interaction of *p*_W_ and *t* (*P* = 0.0654) was also greater than the alpha level, showing that there was no evidence for an interaction between the factors. Thus, the main effects may be interpreted independently of one another: sonication power does not affect stiffness as does only soaking duration. Consequently, the main effects plots showed no appreciable variation of *E* with respect to *p*_W_ (Fig. 7A) and *t* (Fig. 7B).

With regard to the main effects of the respective *p*_W_ and *t* on *σ*_U_, the *P* value (2.32 × 10^−4^) for *p*_W_ was very small showing that there was very strong evidence that sonication power affected σ_U_. On the other hand, the *P* value (=0.194) for *t* was greater than the alpha level, showing that there was no evidence that soaking duration affects *σ*_U_. However, the *P* value (=0.016) for the interaction was smaller than the alpha level, showing that there was evidence for an interaction between the factors. Since interaction was significant, the nonsignificant main effects of *t* in the presence of interaction might well be a result of masking and this lends to the need to observe the influence of *t* at the fixed levels of *p*_W_. As shown in Fig. 8C, with regard to *p*_W_ = 0 and 40%, one-way ANOVA (two-tailed test) revealed that the *P* values for *t* were both greater than the alpha level, showing that there was no evidence that the soaking duration affects σ_U_ at these values of p_W_. However, with regard to *p*_W_ = 20%, the *P* value (=2.05 × 10^−4^) for *t* was very small compared to the alpha level, showing that there was evidence that soaking duration affects *σ*_U_ at this sonication power. In conclusion, with regard to *σ*_U_ the main effects of *t* and *p*_W_ may not be interpreted independently of one another: there is very strong evidence of sonication power having an effect on σ_U_ in general but this may be modified by the soaking duration. From the interaction plot of Fig. 8D and one-way ANOVA (two-tailed test), it was found that increasing *p*_W_ (at *t* = 15 min, and 30 min) resulted in *σ*_U_ peaking at *p*_W_ = 20% but more importantly the *t* = 30 min yielded the higher magnitude of *σ*_U_. However, further increase in *p*_W_ resulted in a decrease in the *σ*_U_. Similarly, the interaction plot of Fig. 8C showed that increasing *t* at only *p*_W_ = 20% resulted in increase in *σ*_U_. In particular, *σ*_U_ peaked at *p*_W_ = 20%. There was no appreciable change in *σ*_U_ with increasing *t* at *p*_W_ = 0 and 40%.

With regard to the main effects of the respective *p*_W_ and *t* on *ε*_U_, the *P* values, i.e., 2.47 × 10^−5^ and 1.95 × 10^−3^, were both very small, showing that there was very strong evidence that both factors affect *ε*_U_. The *P* value (=0.0317) for the interaction was smaller than 0.05, showing that there was evidence for an interaction between the factors. The main effects may not be interpreted independently of one another: there is very strong evidence of sonication power having an effect on *ε*_U_ in general but this may be modified by the extent of the soaking duration. From the interaction plot of Fig. 8F and one-way ANOVA (two-tailed test), it was found that increasing *p*_W_ (at *t* = 15 min, and 30 min) resulted in *ε*_U_ peaking at *p*_W_ = 20% but more importantly the *t* = 30 min yielded the higher magnitude of *ε*_U_. However, further increase in *p*_W_ resulted in a decrease in the *ε*_U_. Similarly, the interaction plot of Fig. 8E showed that increasing *t* at only *p*_W_ = 20% resulted in increase in *ε*_U_. In particular, *ε*_U_ peaked at *p*_W_ = 20%. There was no appreciable change in *ε*_U_ with increasing *t* at *p*_W_ = 0 (albeit a trending increase in the mean value) and 40%. As a mean for comparing with the baseline, the mean *ε*_U_ of untreated specimens was found to be 0.131 ± 0.009 MPa.

Finally, with regard to the main effects of the respective *p*_W_ and *t* on *u*_F_, the *P* values, i.e., 1.58 × 10^−6^ and 2.01 × 10^−3^, were both smaller than the alpha level, showing that there was very strong evidence that both factors affected *u*_F_. The *P* value (=5.12 × 10^−4^) for the interaction was smaller than the alpha level, showing that there was evidence for an interaction between the factors. The main effects may not be interpreted independently of one another: there was very strong evidence of *p*_W_ having an effect on *u*_F_ in general, but this may be modified by the t as shown in Fig. 8. From the interaction plot of Fig. 8H and one-way ANOVA (two-tailed test), it was found that increasing p_W_ (at *t* = 15 min, and 30 min) resulted in *u*_F_ peaking at *p*_W_ = 20% but the *t* = 30 min yielded the higher magnitude of *u*_F_. However, further increase in *p*_W_ resulted in a decrease in the *σ*_U_. The interaction plot of Fig. 8G showed that increasing *t* at only *p*_W_ = 20% resulted in increase in *u*_F_. In particular, *u*_F_ peaked at *p*_W_ = 20%. There was no appreciable change in *u*_F_ with increasing *t* at *p*_W_ = 0 and 40%.

Altogether, the findings from compression testing suggested that 20% sonication power and 30 min soaking time were the optimal conditions resulting in the highest *σ*_U_, *ε*_U_, and *u*_F_.

### Mechanistic analysis of effects of sonication on PLGA and PVA

The intent of this section is to provide a mechanistic analysis to establish simple order-of-magnitude estimates of the sonication energy absorbed by the PLGA and PVA in the scaffold from molecular to bulk level, to help gain insights into the sensitivity of the *u*_F_ to *p*_W_, and *t*.

**Fig. 9:**
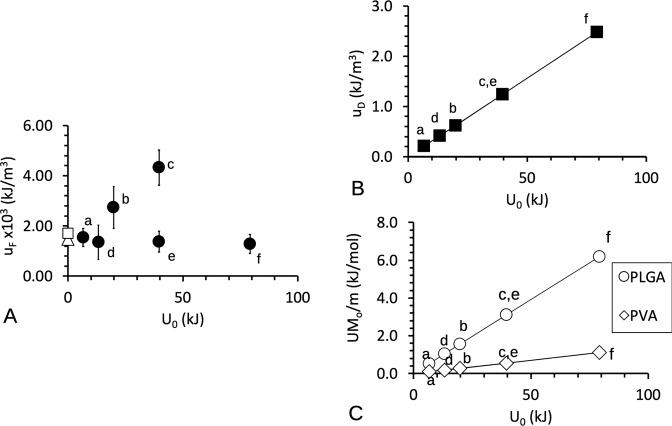
Predictions of the sonication energy absorbed by the PLGA and PVA in the scaffold. Graphs of (**A**) mean scaffold fracture toughness (u_F_), (**B**) predicted strain energy density (u_D_) absorbed by the scaffold to dislodge PVA, and (**C**) predicted energy per mole (UM_o_/m) imparted to the PVA and PLGA polymers, versus the energy generated by the ultrasonic machine at source, *U*_0_. The values of *u*_F_ were derived from Table 2; the error bars represent standard deviation. In (**A**), the two data points (unshaded circle □ and diamond Δ) at *U*_0_ = 0 represent the mean values of the respective untreated scaffold and scaffold treated to soaking only. The letters a, b, and c represent the respective level of (*p*_W_, *t*): (20%, 5 min), (20%, 15 min), (20%, 30 min); d, e, and f represent the respective (40%, 5 min), (40%, 15 min), (40%, 30 min). Symbols *U*, *M*_o_, and *m* represent the bulk energy imparted to the scaffold, the molar mass of the polymer and mass of the polymer, respectively

We begin by inspecting the *u*_F_ at the respective level of *U*_0_ generated by the ultrasonic device. Figure 9A shows a graph of *u*_F_ versus *U*_0_ to illustrate the effect of sonication at bulk level. Each point (*U*_0_, *u*_F_) corresponded to the respective treatment specified by *p*_W_ and *t*, where *U*_0_ was computed using Eq. (1). For comparison, data points (at *U*_0_ = 0) of the mean value of *u*_F_ of untreated scaffold and scaffolds soaked in the solvent mixture in the absence of sonication have been added to the plot. In the absence of sonication, a moderate increase in the *u*_F_ of the treated scaffold (as compared to untreated scaffolds) was observed. This indicated that soaking had a moderate influence in enhancing the stiffness of the scaffold. At low p_W_, a linear increase in the *u*_F_ with increasing *U*_0_ (i.e., by varying the *t*) was observed. This indicated that sonication could result in further enhancement to the *u*_F_ at lower *p*_W_. However, at high *p*_W_, there was no appreciable trending increase or decrease in the *u*_F_ with increasing *U*_0_ (i.e., by varying the *t*). This indicated that sonication had no appreciable influence on the *u*_F_ at higher *p*_W_.

To gain a better understanding of the influence of *p*_W_ on *u*_F_, we present an argument for establishing order of magnitude estimates of the bulk energy absorbed by the scaffold. To begin, we replaced *I* by *U* and *I*_0_ by *U*_0_. For definiteness, the bulk energy imparted to the scaffold, *U*, was found using Eqs. (2) and (3), by setting the μ equal to that of ethanol (=0.0018318 Pa.s, room temperature), γ = 789.30 kg/m^3^ (ethanol), *x* = 120 mm (mid-point of the tub), *f* = 40 kHz, and *G* = 0.016. In forming the scaffold, the molar mass (*M*_o_) of the PLGA and PVA was estimated at 5 and 0.09 kg/mol, respectively. For simplicity, the mass (*m*) of PLGA was assumed to be comparable to that of PVA, valid to order of magnitude (i.e., 10 mg or 10^−5^ kg). The strain energy density absorbed by the scaffold, *u*_D_, was identified with the ratio of *U* to the volume of the scaffold. For the purpose of computing *u*_D_, we estimated the volume of the scaffold as equal to 50 mm^3^. Figure 9B shows a graph of the predicted *u*_D_ versus *U*_0_ to illustrate the predicted energy absorbed by the scaffold. As we might have expected, the *u*_D_ increased linearly with increase in *U*_0_. The predicted values of *u*_D_—estimated to order of magnitude of 10^3^ kJ/m^3^—were much smaller than the *u*_F_ (Fig. 9A). This indicated that the energy that was absorbed by the scaffold was not sufficient to cause the scaffolds to rupture. However, it could have consequential result in dislodging the PVA residuals from the PLGA network.

Let us examine more fundamentally at the molecular level why this should be so. The energy per mole imparted to the respective polymer may be identified with UM_o_/m and is determined as follows. In our simplified model, at a given *t*, the wave propagated through area of order of magnitude of the scaffold surface area. Figure 9C shows a graph of *UM*_o_/*m* imparted to the PVA and PLGA versus *U*_*0*_, to illustrate the effects of sonication at the molecular level. The predictions revealed that increasing *U*_0_ resulted in a linear increase in the *UM*_o_/*m* imparted to the PLGA and PVA respectively. Numerically, the predicted value of the *UM*_o_/*m* ranged 0.1 to 6.0 kJ/mol. Gutowski highlighted that the energy to form interatomic bonds such as covalent bonds ranges 63–920 kJ/mol—derived from basic classical principles of electrostatics and electrodynamics that underpin assumptions about the structure and distribution of the molecules and atoms [23]. Gutowski also highlighted that the energy for van der Waals interactions ranges 4–42 kJ/mol [23]. In our simplified model, the predicted values of *UM*_o_/*m* were much smaller than the energy associated with covalent bonds but were of the same order of magnitude for van der Waals interactions. The latter could contribute to the interaction between PVA residuals and the PLGA network. Thus the energy imparted to the respectively polymer in the scaffold would not be sufficient to cause bond dissociation in the PLGA and PVA molecules but is expected to be able to cause the PVA residuals to be dislodged from the PLGA network.

## Discussion

In this study on a technique of a post-process stage to partially remove the PVA binder in PLGA dental scaffolds, we have investigated the effects of p_W_ and t on the structure and mechanical properties of the PLGA-based scaffold. Our results had enabled us to identify the *p*_W_ and *t* levels that resulted in a scaffold with optimal mechanical properties and structure, as well as a confluence of evidence on the *p*_W_ and *t* central to regulating the amount of PVA present in the scaffold. From FT-IR, it was observed that the amount of PVA decreased with increase in p_W_ and t but this did not completely remove the PVA from the scaffold. From optical microscopy, it was observed that the sonication and prolonged immersion in the solvent resulted in surface unevenness. This was attributed to the shock waves in the form of microscopic jet stream impacting on the scaffold surface (from sonication) and to both processes of dissolution to facilitate PVA removal and the process of emulsification of the PLGA to regain the structure (from the polar-non-polar solvent). The unevenness was least appreciable at *p*_W_ = 20% (22 W) and *t* = 30 min. From the SVR analysis, it was found that high *p*_W_ and *t* resulted in higher surface to volume ratio, which could be attributed to the combined effects of removing PVA (albeit partially) and shrinkage during re-bonding. Results from mechanical testing suggested that there exists a critical value for the respective *p*_W_ and *t* beyond which the scaffold experiences a decrease in fracture strength, fracture strain and fracture toughness. This was found to be *p*_W_ = 20% and *t* = 30 min, which corroborated the findings derived from optical microscopy. The enhanced mechanical properties could be attributed to the overall shrinkage in the scaffold with corresponding reduction in porosity. The mechanistic analysis predicted that the sonication energy imparted to the scaffold was sufficient to disrupt the van Der Waals bonding between the agglomerates of PLGA powder but not high enough to disrupt the covalent bonding within the PLGA. The confluence of evidence revealed an optimal state corresponding to *p*_W_ = 20% and *t* = 30 min whereby the scaffold exhibited enhanced mechanical properties, surface to volume ratio and minimal surface damage in the presence of a reduced PVA concentration.

The mechanical characterization study revealed that reducing the PVA concentration resulted in enhanced mechanical properties which was in good agreement with finding from previous studies. Mechanical characterization of melt-molded scaffolds had revealed that increasing PVA concentration in the PLGA-based scaffold could lead to decreasing elastic modulus [8] and maximum force of rupture [8]. PLGA scaffolds made from wet-spun hollow fiber with porous walls also exhibited decreasing elastic modulus, maximum force of rupture as well as work of fracture with increasing PVA concentration [10]. Altogether these findings are consistent with the rule-of-mixture model for a two-phase composite (Section 2.2), whereby increasing the phase characterized by lower stiffness and strength would result in decreasing the overall stiffness [24] and strength [25] of the composite.

As pointed out in Section 2.1, sonication is known to produce heat that could result in high temperatures at the local regions around the target, i.e., the scaffolds. If the temperatures were sufficiently high, namely 160 °C and above, yellowish tint could appear on the scaffold [15]. This is because when the PVA is heated, it produces polyenes sequences (–CH = CH–)_n_ where the molecules form alternate single and double bonds with a compound group consisting of a carbonyl center and hydrogen [16, 18]. Yet, we did not observe any yellowish tint on the sonicated scaffolds, implying that the local temperatures were not high enough to cause this to happen. To lend support to this conclusion, in the following text, we present an argument for the order of magnitude estimate of the temperature of the surface of the scaffold (*T*_s_). According to Taurozzi et al. the ultrasonic waves could result in bubbles (cavitation process) which when collapsed could generate shock waves, producing liquid jet streaming at speed (*v*) of order of magnitude estimate of 100 m/s. The heat transfer problem may be evaluated to order of magnitude by noting that the *p*_W_ is identified with the rate of heat change, d*Q*/d*t*, where

5 $${{{{{\mathrm{dQ}}}}}}/{{{{{\mathrm{dt}}}}}} \,=\, {{{{{\mathrm{hA}}}}}}({{{{T_s}}}} \,-\, {{{{T_{bath}}}}})$$

where *A* is the surface area of a side face of the scaffold (Section 2.4), *T*_s_ and *T*_bath_ are the temperatures at the scaffold surface and in the bath, respectively, and h is the heat transfer coefficient by convection. We may estimate *h* from

6 $${{{{h}}}} \,=\, {{{{{\mathrm{Nu}}}}}}{{{{.k}}}}/{{{{D}}}}_{{{{{\mathrm{o}}}}}}$$

where *D*_o_ is a characteristic dimension of the target, *k* the thermal conductivity of the solvent, and Nu (the Nusselt number) is given by

7 $${{{{{\mathrm{Nu}}}}}} \,=\, {{{{C}}}}( {{{{{{\mathrm{Re}}}}}}^{{{{m}}}}{{{{{\mathrm{Pr}}}}}}^{1/3}})$$

where Pr is Prandtl’s number, *C* and *m* are constants depending on the value of Re, the Reynold number, given by

8 $${{{\rm{Re}}}} \,=\, \rho {{{{vL}}}}/\mu$$

where ρ is density of the fluid, *v* the speed of the fluid, *μ* the dynamic viscosity of the fluid, and *L* (the dimensional parameter of the Re). At *T*_bath_ = 25 °C, with practical values accorded to the properties of the individual components (ethanol, acetone), namely *ρ* = (785.3 kg/m^3^, 784.0 kg/m^3^); *k* = (0.167 W/m.K, 0.180 W/m.K); *μ* = (0.001074 kg/m.s, 0.000309 kg/m.s) and Pr = (18.05, 4.50), it seems reasonable to identify D_o_ with the thickness of the scaffold wall, i.e., *D*_o_ ∼ *D*_OD_ − *D*_ID_, and *L* with *L*_0_, where the values of *D*_OD_, *D*_ID_, and *L*_0_ (see Section 2.4) were averaged from all the specimens used in the study, for simplicity. Substituting the values of *ρ*, *v*, *L*, and *m* into Eq. (8) to determine Re, at around the scaffold we predicted that the flow attributed to the respective ethanol (Re = 5 × 10^5^) and acetone (Re = 19 × 10^5^) could be turbulent. Noting that *C* = 0.027 and *m* = 0.805, substituting the Re and Pr values into Eq. (7), we obtained the corresponding values of Nu = 3 × 10^3^ (ethanol) and 5 × 10^3^ (acetone). Next, substituting the values of Nu, *k*, and *D*_o_ into Eq. (6), we obtained the corresponding values of *h* = 3 × 10^5^ W/m^2^K (ethanol) and 6 × 10^5^ W/m^2^K (acetone). Finally, substituting the appropriate values for d*Q*/d*t* (=*p*_W_), *h*, *A*_s_, and *T*_bath_ into Eq. (5), we predicted that at the scaffold surface, the ethanol component in the solvent could contribute to raising the temperature from *T*_bath_ to *T*_s_ = 36 °C (22 W) and 46 °C (44 W). On the other hand, the presence of acetone in the solvent could result in *T*_s_ = 31 °C (22 W) and 36 °C (44 W). Of note, the *T*_s_s of the respective ethanol and acetone were smaller than their boiling points, indicating that no boiling had taken place (in reality, only evaporation was observed). More importantly, since these temperatures were lower than the critical temperature (160 °C) for thermal degradation (i.e. resulting in the production of polyene (yellow) residue [16–18]) thus yellowing was not observed during the sonication process. For further details about the calculations and the values used for *ρ*, *k*, *μ*, and Pr, see Supplementary Information.

This technique to enable partial removal of the PVA in the PLGA-based scaffold is promising as it could mitigate several problematic issues related to the PLGA scaffold down the line, such as thermal degradation during sterilization and undue prolong/variability in the biodegradability. There have been many studies on the development and application of PLGA bioscaffolds for dental socket preservation, with and without a binder for the forming process. The use of PVA in the processing of 3D printed PLGA scaffolds is established. However, the process encounters several hurdles when it is translated into commercial production due to regulatory requirements. Moving forward, there are two issues to be addressed before the sonication process can be fully incorporated into the manufacturing process. First, although the presence of PVA in PLGA scaffolds can facilitate the bone regeneration by enhancing the scaffold hydrophilicity (i.e., increasing the concentration of PVA increases hydrophilicity) for cell adhesion and growth on the scaffold [8], the possibility of toxicity arising from changes to the polymers during sonication and solvent immersion may not be ruled out. This would require carrying out cell viability test, e.g., to assess for cytotoxicity to osteoblasts by observing for proliferation. This has been identified as an area for further study. The second issue concerns the sterilization stage. Sterilization could involve one or more of the following, namely heat, high pressure and radiation [26], which could result in thermal degradation of the PVA. As pointed out earlier with regard to previous studies, under heat treatment at high temperatures, decolorization (i.e., yellowing) was observed due to the presence of polyene residues; irradiating the scaffolds could result in radiation-induced cross-links. Clearly reducing the presence of PVA would minimize this effect—and in turn mitigate the effects on the mechanical properties—which was what the current study aimed to establish. An investigation into how sterilization affects the mechanical properties of the scaffolds that were treated to the sonication process was out of the scope of this study. However, this had also been identified as an area for future study.

## Conclusions

We have proposed a post-processing technique to partially remove the PVA in the PLGA/PVA scaffold by sonicating the PLGA/PVA scaffold in the presence of a solvent mixture possessing polar-nonpolar properties. The effects of *p*_W_ and *t* on the structure (SVR) and mechanical properties (*E*, *σ*_U_, *ε*_U_, and *u*_F_) of PLGA/PVA scaffolds immersed in the ethanol/acetone as the polar-nonpolar solvent mixture were investigated. The findings from this study are summarized as follows.

- The optimal state of the scaffold, associated with the highest *σ*_U_, *ε*_U_, and *u*_F_, occurred at *p*_W_ = 20% and *t* = 30 min, which corroborated the finding of minimal surface unevenness as seen from optical microscopy.
- FT-IR showed that the PVA was not completely removed from the PLGA scaffolds over the range of soaking duration and sonication power investigated.
- A mechanistic analysis of the energy imparted to the respectively polymer in the scaffold indicated that the energy would not be sufficient to cause bond dissociation in the PLGA and PVA molecules but is expected to be able to cause the PVA residuals to be dislodged from the PLGA network.

This simple technique is promising as it can help to regulate the amount of PVA, i.e., partially removing PVA, that is present in the PLGA scaffold and to optimize the scaffold toward achieving enhanced *σ*_U_, *ε*_U_, and *u*_F_ within the regulatory controls.

## Supplementary information

Supplementary Information

## Data Availability

The data that support the findings of this study are available from the corresponding author, KLG, upon reasonable request.
