# Supplementary material for: Post-process optimization of 3D printed poly(lactic-co-glycolic acid) dental implant scaffold for enhanced structure and mechanical properties: effects of sonication duration and power
Source: J Mater Sci Mater Med. 2021 Jul 31;32(8):91. doi: 10.1007/s10856-021-06561-3 (PMC8325663; doi:10.1007/s10856-021-06561-3)
Supplement: Supplementary file 1 — Supplementary Information [file 10856_2021_6561_MOESM1_ESM.docx]

SUPPLEMENTARY INFORMATION

**Post-process optimization of 3D printed poly(lactic-co-glycolic acid) dental implant scaffold for enhanced structure and mechanical properties: effects of sonication duration and power**

R. N. V. C. Virinthorn, M Chandrasekaran, K Wang, K. L. Goh

# Highlights from the literature

Table SI- 1. Findings from previous studies of the mechanical properties and structure of PLGA-based scaffold that utilized PVA as a binder

| **Porous scaffold forming method** | **Force-displacement properties** | **Stress-strain properties** | **Comments** | **Ref** |
| --- | --- | --- | --- | --- |
| Melt-molding particulate-leaching to produce sponge scaffold | 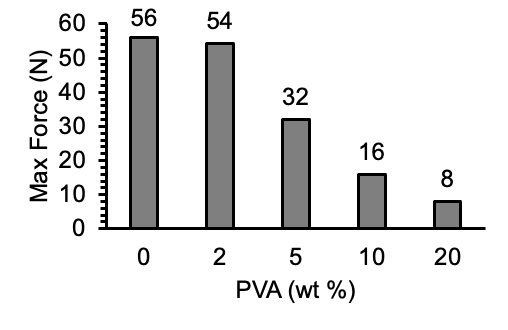  Graph of maximum force versus PVA concentration under biaxial tensile testing. Maximum force (N) was derived from kgf/mm,  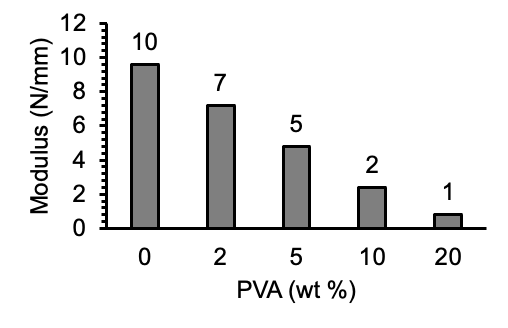  Graph of elastic modulus versus PVA concentration, under biaxial tensile testing. Elastic modulus (N/mm) was derived from (kgf/mm)/mm. | N/A | - In general, there was a trending decrease in Maximum force (to rupture) and Elastic modulus, with increasing PVA concentration. - Notably, at PVA = 20 wt%, the maximum force was 7 times lower than the control (0 wt%); the modulus was 10 times lower than the control. - Scaffolds were highly porous from interior to surface; pore size ranges 200–300 mm; porosity, about 90%, almost independent of PVA concentration (0-20%) | [1] |
| Wet spinning, to produce PLGA hollow porous fibre | 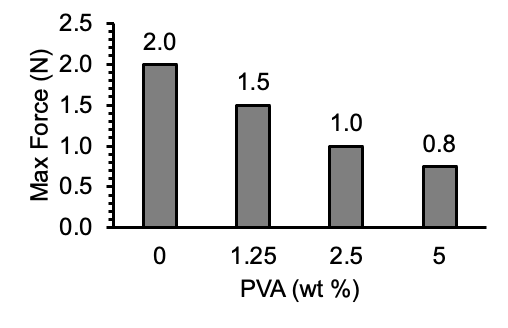  Graph of maximum force versus PVA concentration. The maximum force was derived from representative plots of force versus displacement.  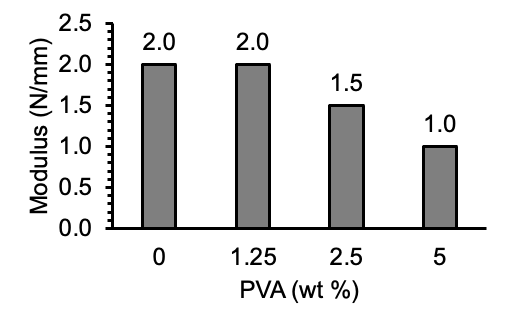  Graph of Stiffness versus PVA concentration. The stiffness was derived from linear region of representative plots of force versus displacement.  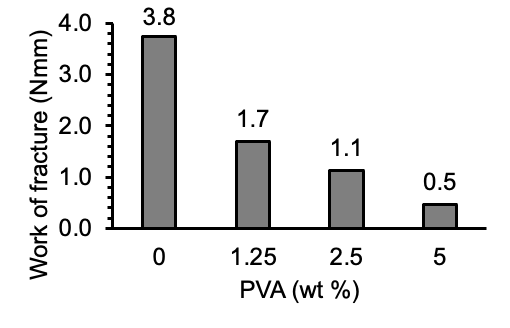  Graph of Work of fracture versus PVA concentration. The Work of fracture was derived from estimates of area under the curve of representative plots of force versus displacement. | 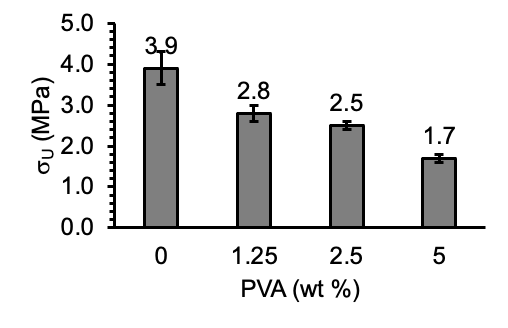  Graph of Fracture strength versus PVA concentration derived from tensile testing  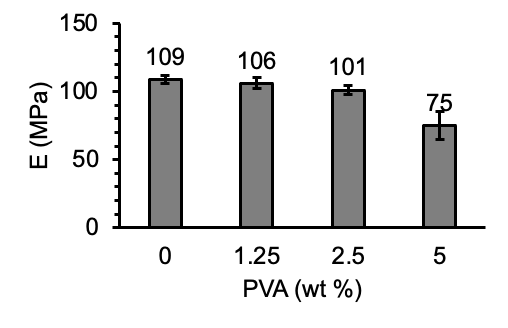  Graph of Stiffness versus PVA concentration derived from tensile testing | - Similar to Oh et al. [1], for the force-displacement curve, there was a trending decrease in Maximum force (to rupture) and Elastic modulus, with increasing PVA concentration. - Notably, the control exhibited a maximum force 2.5 times higher than that at PVA = 5 wt%, modulus of 2 times higher than that at PVA = 5 wt%, and work of fracture 7.6 times higher than that at PVA = 5 wt%; - For the stress-strain curve, there was a trending decrease in fracture strength σ_U_ and stiffness E, with increasing PVA concentration. - However, the decrease in σ_U_ (MPa) and E (MPa) were more gradual than Maximum force (N) and Modulus (N/mm). Notably the control exhibited a σ_U_ 2.3 times higher than that at PVA = 5 wt%, and E 1.5 times higher than that at PVA = 5 wt%, - The presence of PVA significantly increased the pore size and overall porosity in PLGA fibres (i.e. 0% versus 1.25%, 2%, 5% PVA concentration) | [2] |
| Emulsion electrospinning to produce nanofibrous mat-like scaffold | N/A | 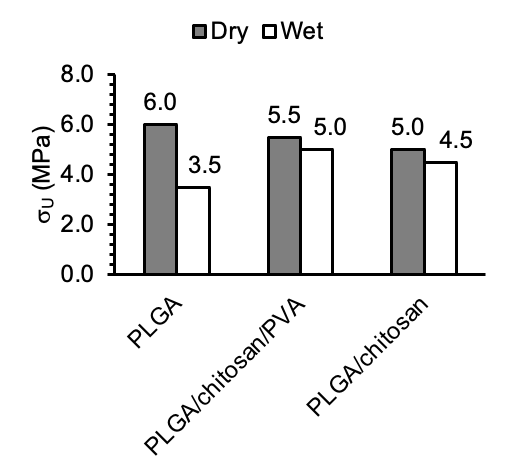  Graph of Fracture strength (σ_U_) versus PVA concentration derived from tensile testing. The values were taken from the maximum stress of representative plots of stress versus strain.  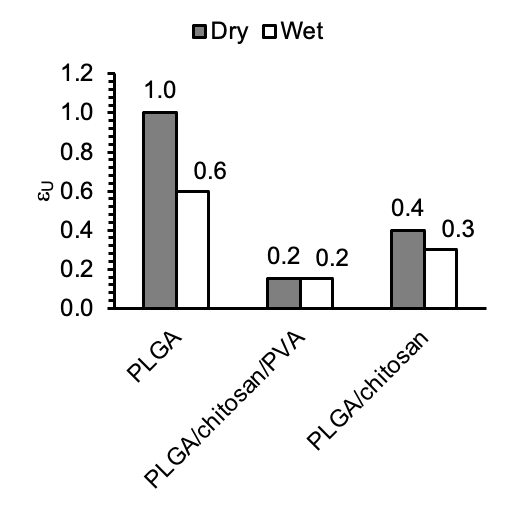  Graph of Fracture strain (ε_U_) versus PVA concentration derived from tensile testing. The values were taken from the maximum strain of representative plots of stress versus strain.  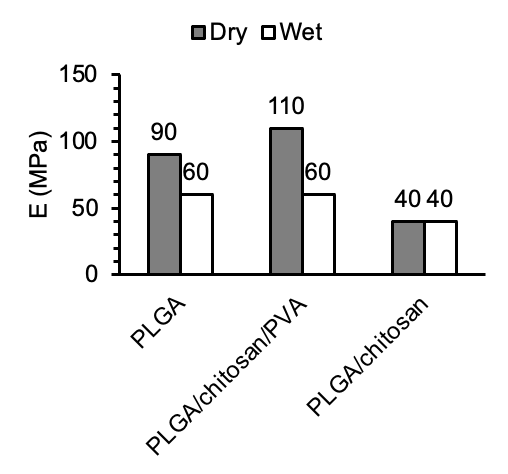  Graph of Stiffness (E) versus PVA concentration derived from tensile testing. The values were taken from calculating the slope of the linear portion of representative plots of stress versus strain.  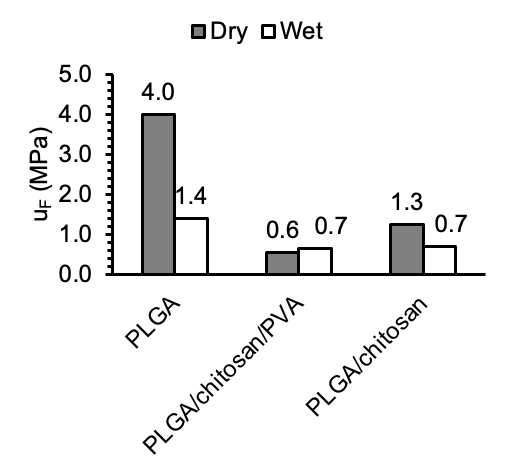  Graph of Fracture toughness (u_F_) versus PVA concentration derived from tensile testing. The values were taken from calculating the area under the curve of the representative plots of stress versus strain. | - There are three important points: (1) the composite (i.e. PLGA/Chitosan/PVA or PLGA/Chitosan) fibrous mat exhibited reduced magnitude in the mechanical properties (except for E) compared to PLGA. (2) Dry specimens exhibited enhanced mechanical properties compared to wet specimens. (3) There is no clear advantage in the mechanical property of PLGA/Chitosan nanofibers formed from PLGA/Chitosan/PVA nanofibers treated to ethanol to extract PVA. For instance, while strength remained relatively unchanged, the PLGA/Chitosan exhibited enhanced extensibility (ε_U_) and toughness but lower stiffness compared to PLGA/Chitosan/PVA. - The diameter of slectro-spun fibre decreased with increasing PVA concentration. | [3] |

# Order of magnitude estimates of composition of pristine PLGA/PVA scaffold

Details of the method for processing the scaffold was proprietary information. However the composition of the components of the PLGA-based scaffold could be estimated based on the information derived from the patent document [4] informed by basic design concepts of composite materials and foams. For the benefit of the reader, this section provides simple order-of-magnitude estimates of the composition of the PVA and PLGA used. The PLGA/PVA scaffold is a two-phase composite whereby the stiffness (E_C_) of the PLGA/PVA composite material depends on the elastic moduli, E_PLGA_ and E_PVA_, and volume fractions, V_PLGA_ and V_PVA_, of the respective PLGA and PVA, which could be determined valid to order-of-magnitude using the simple rule-of-mixture [5], i.e. E_C_ = E_PLGA_V_PLGA_ + E_PVA_V_PVA_. The basic approach to designing the scaffold to a desirable stiffness required knowledge about the elastic moduli. With representative values E_PLGA_ = 2.4 GPa [6] and E_PVA_ = 1.4 GPa [7], it seemed reasonable to take V_PLGA_ ≈ 0.6 and V_PVA_ ≈ 0.4, for blending two polymers such that the polymer with the larger amount provides the structural support. From the simple rule-of-mixture, we thus predicted that, to order of magnitude, E_C_ = 2.0 GPa. The stiffness (E_S_) of the porous scaffold depends on the porosity. In general E_S_ would be much lower than E_C_. The E_S_ could be estimated using the Gibson and Ashby model given by

| E_S_ = λE_C_(ρ_S_/ρ_C_)^n^, | (1) |
| --- | --- |

where ρ_S_ and ρ_C_ denote the densities of the respective scaffold and the equivalent solid material, and λ and n are constants that depend on the foam microstructure [6]. Thus, ρ_S_/ρ_C_ parameterizes the porosity of the scaffold with values falling between 0 and 1. With practical values n = 3.5 (assuming an open-cell porous scaffold) and λ = 1 for PLGA structures [6], it seemed reasonable to take ρ_S_/ρ_C_ ~≈ 0.6. Consequently, we found that the E_S_ = 0.19 GPa (or 190 MPa), in good order-of- magnitude agreement with the empirical value of the untreated PLGA/PVA scaffolds, i.e. 171.9±53.0 MPa (Manuscript, Table 2). Thus we showed that the amount of PVA and PLGA used in terms of volume fraction were 0.4 and 0.6, respectively, valid to order of magnitude. Consequently, the volume ratio of PVA:PLGA (=2:3) was also within the range (namely within 2:8 to 7:10) provided by the PLGA scaffold manufacturing company.

# Thermal analysis

Table SI- 2. Properties^#^ and derived parameters of the ethanol and acetone liquids at 25 ^o^C.

|  | Speed of wave during collapse of bubble, v (m/s) [8] | Prandtl’s number, Pr | Density,ρ kg/m^3^ | Thermal conductivity, k, W/m.K | Dynamic Viscosity, μ kg/m.s | Reynold’s number  Re (=ρvL_0_/μ) | Nuselt’s number, Nu | Heat transfer coefficient for convection h=Nu*k/Dx | Temperature of surface of scaffold Ts ^o^C (@22 W) | Temperature of surface of scaffold Ts ^o^C (@44 W) |
| --- | --- | --- | --- | --- | --- | --- | --- | --- | --- | --- |
| Ethanol | 100 | 18.05 | 785.3 | 0.167 | 0.001074 | 5x10^5^ | 3x10^3^ | 3x10^5^ | 36 | 46 |
| Acetone | 100 | 4.5* | 784 | 0.180 | 0.000309 | 19x10^5^ | 5x10^3^ | 6x10^5^ | 31 | 37 |

^#^Unless otherwise stated, the values of the properties were obtained from Cengel [9] and engineering toolbox (www.engineeringtoolbox.com)

* Prandtl’s number of acetone at 25 ^o^C was identified to order of magnitude.

# Order of magnitude estimates of post-sonication PVA volume fraction using Beer-Lambert law

In this section, we described how we applied models based on the classic Beer-Lambert Law to compute order-of-magnitude estimates, of the volume fractions of PVA left in the scaffold.

The concentration of functional groups of the PVA may be estimated to order of magnitude by replacing the functional layer thickness by the specimen layer thickness, giving

| C_x_ = A_x_/[et] | (2) |
| --- | --- |

where C_x_ is the concentration of the functional group (units: mol.cm^-3^), A_x_ represents the intensity of the absorbance of the functional group, e represents the molar extinction coefficient of the functional group (units: cm^2^.mol^-1^) and t is the layer thickness (units: cm). Subscript x corresponds to p_W_ at 0, 20% and 40% (e.g. A_20_ and C_20_ referred to the absorbance intensity and concentration at p_W_=20%).

The remaining amount of PVA present in the sonicated scaffold can be determined by defining the relative concentration of C_x_, i.e. with respect to C_0_, as

| C_x_/C_0_ = A_x_/A_0_, | (3) |
| --- | --- |

and by noting that C_x_ is proportional to the normalized absorbance A_x_’, where

| A_x_’ = A_x_/A_max_, | (4) |
| --- | --- |

and A_max_ is the absorbance intensity corresponding to the difference between the highest and lowest intensity points, which could be observed in the wavenumber range 1730-1930 cm^-1^.

From Figure 4, it was found that A_max_ = 3.55 (p_W_=0), 3.00 (p_W_=20%) and 2.31 (p_W_=40%). With a practical choice of the absorbance intensity (A_x_) at wavenumber = 1683 cm^-1^ contributed by the C-O in the R-O-H of PVA and C=O group of the polyvinyl acetate, we evaluated the intensity difference between the lowest absorbance intensity (i.e. the highest point, which occurred within the range 1730 cm^-1^ to 1930 cm^-1^) and the absorbance intensity at 1683 cm^-1^. We then found that A_0_ = 3.05, A_20_=2.01, and A_40_=1.31. We substituted these values into Eq. (4) to find that, to order of magnitude, A_0_’ = 0.86, A_20_’ = 0.67 and A_40_’ = 0.57. Consequently we found, through the use of Eq. (3), that C_20_/C_0_ = A_20_’/A_0_’ = 0.78 and C_40_/C_0_ = A_40_’/A_0_’ = 0.66.

Thus the amount of PVA residue present in the sonicated scaffold was 0.78 (p_W_=20%) times and 0.66 (p_W_=40%) times smaller than that of the pristine scaffold. Recalled that the volume fraction of PVA in the pristine scaffold, V_PVA_ ~ 0.4 (section 2.2), it follows that the volume fractions of the PVA residue in the sonicated scaffold treated at p_W_ = 20% and 40% were of the order of V_PVA_ times C_20_/C_0_ and C_40_/C_0_, respectively. We therefore predicted that, to order of magnitude, the volume fraction of PVA decreased to (i) 0.31 (p_W_=20%) and (ii) 0.26 (p_W_=40%). Overall, this indicated a progressive decrease in PVA with increasing p_W_.

# Examination of PLGA-based scaffold texture

Photos of sonicated versus non-sonicated scaffold are presented in Figure SI-1. These photos depict the macroscopic and microscopic views of fractured PLGA scaffolds, to reveal the texture at different areas of the scaffold, namely the end faces, the inner surfaces and the cross section through the scaffold.

Here, the photos (Figure SI-1 A-D) from the camera (which had a wide depth-of-field) facilitated the display of the overall texture of the specimen when seen under a fairly uniform illumination. In all cases of the photos taken, no dark shades were observed at the macroscopic level.

As for the microscopic images taken using optical microscopy, these images (Figure SI-1 E, F) were taken at a higher magnification than those shown in the main text (Figure 3). The bright and dark features observed revealed the undulating surface, made visible by the direction of the light rays. Any chemical changes, if any, clearly did not produce any change in shade that could be observable under the light microscope.


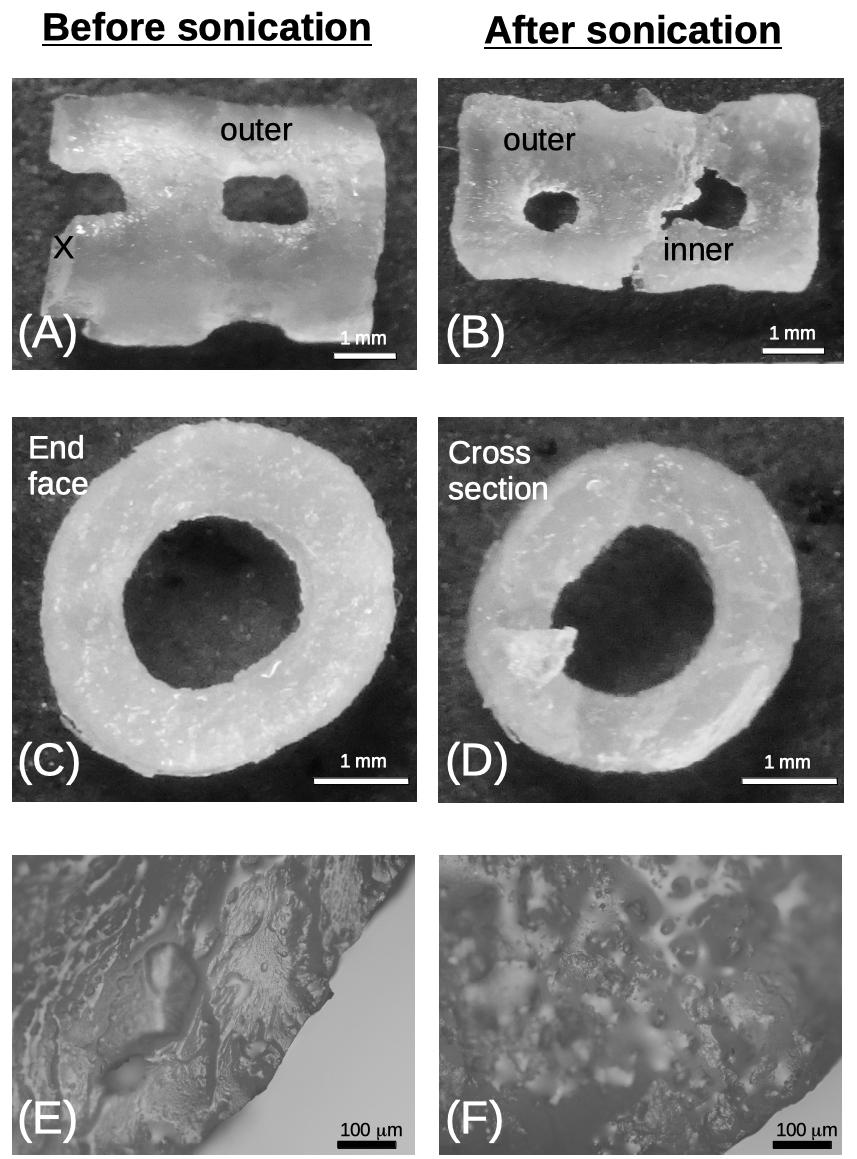


Figure SI-1. Images of the PLGA scaffolds. (A-D) Macroscopic view of the scaffold. Images were taken using a camera (Olympus TG870). (E-F) Microscopic view (in bright field mode). The images were taken using the Olympus microscope (BX51M). In panel D, ‘Cross section’ of the scaffold is revealed for the crack surface shown. In panel A and B, ‘outer’ and ‘inner’ refer to the outer and inner surfaces of the scaffold. Also, in panel A, symbol ‘X’ indicates a crack surface showing the cross section of the scaffold

# References

1. Oh, S.H., et al., *Fabrication and characterization of hydrophilic poly(lactic-co-glycolic acid)/poly(vinyl alcohol) blend cell scaffolds by melt-molding particulate-leaching method.* Biomaterials, 2003. **24**: p. 4011-4021.

2. Meneghello, G., et al., *Fabrication and characterization of poly(lactic-co-glycolic acid)/polyvinyl alcohol blended hollow fibre membranes for tissue engineering applications.* Journal of Membrane Science, 2009. **344**(1-2): p. 55-61.

3. Ajalloueian, F., et al., *Emulsion electrospinning as an approach to fabricate PLGA/chitosan nanofibers for biomedical applications.* BioMed Research International, 2014. **Volume 2014**: p. 13.

4. Chandrasekaran, M., S.X. Zhang, and B.Y. Tay, *Method for obtaining graded pore structure in scaffolds for tissues and bone and scaffolds with graded pore structure for tissue and bone*, T.R.o.P. Singapore, Editor. 2005, Agency for Science, Technology and Research: Singapore.

5. Xie, J.Z., et al., *Influence of hydroxyapatite crystallization temperature and concentration on stress transfer in wet-spun nanohydroxyapatite-chitosan composite fibres.* Biomedical Materials, 2008. **3**: p. 2-6.

6. Ebrahimian-Hosseinabadi, M., et al., *Evaluating and Modeling the Mechanical Properties of the Prepared PLGA/nano-BCP Composite Scaffolds for Bone Tissue Engineering.* Journal of Materials Science & Technology, 2011. **27**(12): p. 1105-1112.

7. Parthasarathy, V., et al., *Evaluation of mechanical, optical and thermal properties of PVA nanocomposites embedded with Fe2O3 nanofillers and the investigation of their thermal decomposition characteristics under non-isothermal heating condition.* Polymer Bulletin, 2020.

8. Sahoo, S.K., et al., *Residual polyvinyl alcohol associated with poly (D,L-lactide-co- glycolide) nanoparticles affects their physical properties and cellular uptake.* Journal of Controlled Release, 2002. **82**: p. 105-114.

9. Cengel, Y.A., *HeatTransfer-A Practical Approach*. 2002, McGraw-Hill. p. 896.
